# Prognostic factors in neuroendocrine carcinoma: biological markers are more useful than histomorphological markers

**DOI:** 10.1038/srep40609

**Published:** 2017-01-11

**Authors:** Patricia Freis, Emmanuelle Graillot, Pascal Rousset, Valérie Hervieu, Laurence Chardon, Catherine Lombard-Bohas, Thomas Walter

**Affiliations:** 1 Hospices Civils de Lyon, Hôpital Edouard Herriot, Service Central d’Anatomie et Cytologie Pathologiques, 69437 Lyon cedex 03, France; 2 University of Lyon, Université Lyon 1, France; 3 Equipe Signalisation de l′Immunité Innée et Oncogénèse, Centre de Recherche en Cancérologie de Lyon, UMR Inserm 1052 CNRS 5286, 69373 Lyon cedex 08, France; 4 Hospices Civils de Lyon, Hôpital Edouard Herriot, Service de Gastroentérologie et d’Oncologie Digestive, 69437 Lyon cedex 03, France; 5 Hospices Civils de Lyon, Hôpital Edouard Herriot, Radiologie, 69437 Lyon cedex 03, France; 6 Hospices Civils de Lyon, Hôpital Edouard Herriot, Service de Biochimie, 69437 Lyon cedex 03, France

## Abstract

Gastroenteropancreatic neuroendocrine carcinomas (GEP-NEC) are a very aggressive type of cancer, for which prognostic factors are lacking. We analysed clinical and histomorphological prognostic markers of overall survival (OS), completed with a record of biological and haematological data of patients diagnosed between December 2002 and December 2015. The median OS was 16 months (95% CI 13.9–18.1). After univariate analysis, performance status (PS) ≥ 2 and stage IV were associated with a worse outcome (9 months and 14 months, respectively), as well as patients with lactate dehydrogenase (LDH) and aspartate aminotransferase (AST) levels ≥ 2 ULN (9 months and 8 months, respectively). After multivariate analysis, LDH and AST levels were the only factors that remained significantly associated with better survival: HR 0.36 (*p* = 0.04) and 0.31 (*p* = 0.03), respectively. When patients had elevated LDH and AST levels, OS was 20 months, when they had high LDH or AST levels, 13 months and 8 months in the group with low LDH and AST levels (*p* < 0.001). Therefore, biological data appeared to be more relevant prognostic factors than usual factors described in other studies (PS, stage, and Ki-67). Considering LDH and AST levels at diagnosis could help physicians to predict survival and to stratify patients for clinical trials.

Gastroenteropancreatic neuroendocrine carcinomas (GEP-NEC) represent less than 1% of digestive cancers and 7 to 21% of neuroendocrine neoplasms^1^. In the 2010 World Health Organisation (WHO) classification, NEC are defined as poorly differentiated tumours, with small or large cells, expressing neuroendocrine markers chromogranin A (CgA) and synaptophysin, and with a high proliferative index (grade 3 with a Ki-67 > 20%). NEC prognosis is poor, as patients usually present with metastatic disease at diagnosis (more than 80% of patients) and because of the relative lack of effective therapy. Prognosis varies according to disease stage at diagnosis. Based on Surveillance, Epidemiology, and End Results (SEER) data for 2546 patients with gastrointestinal NEC, Sorbye *et al*. evaluated that the median survival of patients with localized disease was 38 months, of those with regional disease 16 months, and of those with distant disease 5 months^2^. To date, prognostic survival factors have rarely been explored in NEC. Some authors have investigated biological data for use as prognostic factors in neuroendocrine tumour (NET) and NEC patients, and these have included lactate dehydrogenase (LDH)^3^,^4^, CgA^3^,^4^, neuron-specific enolase (NSE)^4^, platelets, haemoglobin, white blood cell count, alkaline phosphatase (ALP), and C-reactive protein (CRP)^3^. Several studies in other aggressive types of cancer have described biological characteristics as prognostic tools, such as LDH and ALP levels in small cell lung cancer^5^,^6^, or neutrophil-to-lymphocyte ratio (NLR) in biliary tract and gastric cancers^7^,^8^. Recent studies have also reported the potential of blood transcript analysis as a predictive and prognostic marker of progression in well-differentiated NET^9^,^10^, but this was not studied in NEC patients. Moreover, molecular analysis, such as p53 and retinoblastoma (RB) protein staining, are probably promising in NEC^11^, but these analyses are not validated for survival prognosis in NEC. In this context, we conducted a retrospective study to evaluate usual relevant clinical and histomorphological prognostic markers of overall survival in patients with NEC, completed with biological and haematological data, which are convenient and directly available to physicians.

## Results

### Patient characteristics

A total of 109 patients referred to the Edouard Herriot hospital (Lyon, France) between 2002 and 2015 for GEP-NEC were identified. Nine patients were excluded because of well-differentiated NET (n = 6) or because their medical file was not available (n = 3). Therefore, 100 patients were included in the study.

The median age at diagnosis was 64 years (mean 63 years, range 30–89 years) and 67% were male. The most frequent primary tumour locations were: duodenum-pancreas (30%), colon-rectum (26%), unknown location (24%), and oesophagus-stomach (15%). Among the symptoms at diagnosis pain (57%) and weight loss (40%) were common features, whereas bowel disorders (20%), icterus (13%), and mass syndrome (6%) were less frequently reported. Thirty-one percent had an Eastern Cooperative Oncology Group Performance Status (ECOG-PS) ≥ 2. Only 1 patient presented a functioning tumour with adrenocorticotrophic hormone (ACTH) secretion. The majority of patients had stage IV disease (81%), defined by the presence of at least 1 metastatic site. The most common metastatic sites were the liver (56%) and distant lymph nodes (50%). Somatostatin Receptor Scintigraphy (SRS) was performed in 24 patients (24% of the population), and was positive in 48% of these; fluorodeoxyglucose positron emission tomography (FDG-PET) was performed in 46 patients (46% of the population) and was positive in 91% of these. Primary tumour resection was performed in 28% of the population (Table 1), and 42% of them presented a stage I-III tumour. All patients received standard chemotherapy regimens for NEC (cisplatin or carboplatin with etoposide), as first-line palliative chemotherapy or in curative intent with surgery or radiotherapy for stage I-III NEC.

Increased (≥2 upper limit of normal, ULN) levels of CgA were found in 41% of the 44 patients with data, increased NSE was found in 59% of the 44 patients with data, and LDH in 29% of the 52 with data. Among the 15 patients with an elevated LDH level (Table 2), 60% presented liver metastasis. The median ALP level was at the ULN. Increased AST levels were found in 19/61 of those with data (31%; Table 2), among whom 89% had liver metastasis. Thirty percent of patients with liver metastasis had an elevated AST level, and only 4% patients without liver metastases had an elevated AST level. The median values for complete blood cell count and coagulation factors (fibrinogen, prothrombin time – PT, activated partial thromboplastin time – aPTT) were normal, although fibrinogen which was near the ULN (Table 2).

Histological analysis was performed on primary tumour site (63%) or on metastatic site (36%). Large cell NEC was the most prevalent morphology (59%). Synaptophysin staining was positive for almost all patients (98%) with data, and CgA staining was positive nearly three-quarters (72%) of those with data. The median level of Ki-67 index was 70% (20–100%). Necrosis status was recorded in 58 patients, and 86% of them were positive (Table 3).

### Overall survival and prognostic factors

**Figure 1:**
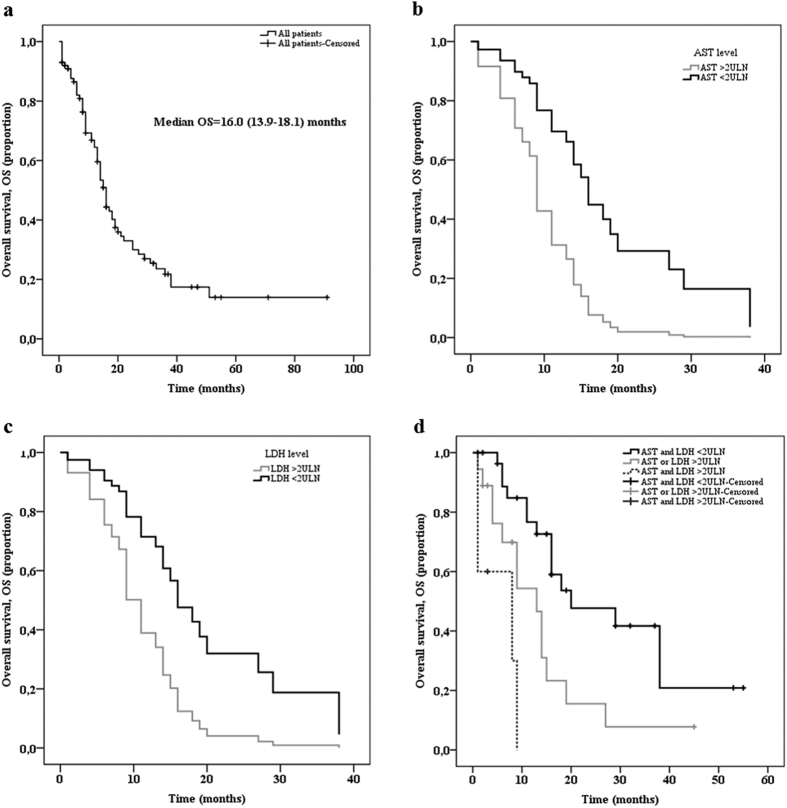
Overall survival (OS) among the population with GEP-NEC (**a**), and according to serum AST level (**b**), serum LDH level (**c**), and combining AST and LDH (**d**).

The median (range) duration of follow-up was 13 (1–91) months and the median overall survival (OS) was 16 months (95% confidence interval – CI 13.9–18.1; Fig. 1a). Among the 66 deceased patients, 63 died of the cancer and 3 had no known cause of death; the cancer specific survival and OS were the same (median of 16.0 months). After univariate analysis, the following clinical, biological, and morphological factors were associated with shorter OS: ECOG-PS ≥ 2, stage IV disease (Table 1), LDH ≥ 2 ULN, and AST ≥ 2 ULN (Table 2). The other factors investigated, such as inflammation markers (low albumin, high CRP, and high NLR ratio) were not prognostic factors. Patients with a Ki-67 < 55% (median OS: 22 months; 95% CI 10.2–33.8) had a longer OS than patients with a Ki-67 ≥ 55% (median OS: 14 months; 95% CI 12.0–16.0), but this difference was not significant (*p* = 0.06; Table 3). Primary tumour resection was associated with better median OS (25 months, 95% CI 14.1–35.9), compared to non-resected patients (13 months, 95% CI 11.4–14.6; *p* = 0.004; Table 1), but was not included in the multivariate analysis because this treatment is not a prognostic factor present at diagnosis. The metastases resection had no impact on OS (Table 1).

Factors found to be significantly associated with OS in univariate analysis were tested in multivariate analysis, namely ECOG-PS, stage, AST, and LDH levels. As shown in Tables 1 and 2, ECOG-PS data was available for 64 patients, stage data for all patients, AST data for 61, and LDH data for 52 patients. Multivariate analysis was performed on the 39 patients without missing data. Median OS for these patients (15 months, 95% CI 11.9–18.1) was not significantly different to that of the 61 patients with at least one missing data (16 months, 95% CI 12.7–19.3; *p* = 0.51). Cox regression found that elevated LDH (hazard ratio – HR: 0.36, 95% CI 0.13–0.97; *p* = 0.04) and AST (HR: 0.31, 95% CI 0.11–0.91; *p* = 0.03) levels were significantly associated with better survival (Table 4, and Fig. 1b,c). After stratification of patients according to median OS, 3 groups of patients were clearly separated into prognosis groups. The OS was 20 months (95% CI 3.9–36.1) when neither AST or LDH levels were ≥ 2 ULN, 13 months (95% CI 7.6–18.4) when AST or LDH levels were ≥ 2 ULN, and 8 months (95% CI 0.0–19.0) when AST and LDH levels were ≥ 2 ULN (*p* < 0.001; Fig. 1d).

## Discussion

The aim of this study was to analyse several prognostic factors in a GEP-NEC cohort, including a review of biological factors in addition to other characteristics already identified at baseline in this tumour type, namely clinical, morphological, and histological factors. Searching for additional prognostic factors is essential in NEC, as clinicians lack tools to identify patients who may have longer survival and therefore may benefit more than one line of treatment and/or inclusion in clinical trials.

In multivariate analysis, adjusting for variables including ECOG-PS and stage, AST and LDH levels independently predicted the OS of patients with NEC. If confirmed by other studies, stratification of patients based on AST and LDH levels could help physicians to predict survival and separate patients into groups for clinical trials. In the present study, median OS for patients with high AST and LDH levels, high AST or LDH levels, and low AST and LDH levels differed significantly. The NORDIC study, performed in 12 Nordic University Hospitals and 308 eligible patients, also demonstrated that elevated LDH level was a negative prognostic factor^3^, as well as a study on 100 colorectal NEC performed in a single cancer centre in Texas^12^. Both studies did not analyse all liver enzymes, as we did in the present study. This is therefore the first report of an association between elevated transaminase and poor prognosis. A high LDH level can be explained by the reliance of tumour cells on increased glycolysis that results in increased lactate production instead of aerobic respiration in the mitochondria, even under oxygen-sufficient conditions (a process also known as the Warburg effect)^13^. As NEC positively respond to FDG-PET they consume large quantities of glucose and therefore produce more lactate than normal cells. Moreover, these tumours are poorly vascularized and highly proliferative^14^, two factors that could promote hypoxia within tumours. These conditions (high consumption of glucose and hypoxic environment) lead to higher LDH levels. More generally, a high LDH level is known to be a factor of poor prognosis in other cancers, such as lung^15^ and breast cancer^16^, and two other studies in NEC (pulmonary^17^ and colorectal^12^) also reported high LDH serum levels. Among 61 patients with available AST data at diagnosis, 31% had AST ≥ 2 ULN and they were associated with poorer outcome in multivariate analysis. The rise of AST serum levels is, at least in part, explained by liver involvement as the proportion of patients with high AST levels was 31% among those with liver metastases *versus* 4% in those without liver metastases.

Very recently, one study performed on 149 pancreatic neuroendocrine neoplasms demonstrated that CRP level is a new prognostic factor for survival^18^. This is, to date, the only study that analysed an inflammatory marker as prognostic tool in neuroendocrine neoplasms. The CRP/albumin ratio is also a prognostic factor for survival in small cell lung cancer^19^. Based on these studies, we analysed CRP and albumin levels in the present NEC cohort, however neither CRP nor albumin levels were prognostic factors in this population. The NLR reflects the immune status of patients and a high NLR is associated with poorer survival in biliary tract cancer^8^ and in gastric cancer^7^. After analysis of NLR with the two cut-off levels used in other studies, neither a cut-off value of 3 or 4 were prognostic factors in this population. There was a trend towards worse survival for those with a NLR ≥ 4, and it will be interesting to further explore the NLR in a larger NEC cohort to see whether this factor is a useful prognostic tool.

Among histological characteristics, and accordingly to other studies, there was no difference between small or large cell tumours in the present study^3^,^20^,^21^,^22^. The NORDIC study described the cut-off of 55% for Ki-67 as a prognosis factor for NEC^3^, we therefore analysed OS accordingly and patients with a Ki-67 above 55% did have a shorter OS, but this difference was not significant. Primary tumour resection but not metastatic resection, was associated with better OS after univariate analysis and remains an option for the treatment of local NEC.

The limitations of this study are the ones that inherently apply to population data which are collected retrospectively. Multiple comparisons is a limit in this study. However, after Bonferroni adjustment, LDH and AST levels remained significant parameters (*p* < 0.001), whereas ECOG-PS and stage were not (*p* = 0.02). This study was performed on a small number of patients, due to the rarity of the disease and compared to other studies performed in multiple institutions. Biological data were not available for all patients, which decreases the power of the multivariate analysis. However, the median OS between the group of patients with all data available and the group with at least one missing data was similar. In addition, all patients were treated, and baseline characteristics presented were collected around the date of diagnosis (+ /−2 weeks) before the beginning of any treatment. We therefore cannot study whether biomarkers (LDH, AST) are only prognostic factors or whether they are also predictive factors of response to systemic chemotherapy (platinum-etoposide), which was the standard first-line treatment. Nevertheless, an important strength of this study is that biological tests were performed in a single institution, and are therefore more homogeneous. It is also of note that this study is one of the largest performed in a single institution (109 patients over a 13-year period, which compares, for instance, to 100 patients with colorectal NEC who were reviewed over a 22-year period in a single centre and reported by Conte *et al*.^12^.

In conclusion, we report herein that patients could be stratified into 3 groups according to a combination of LDH and AST levels which can help physicians to predict survival and to choose patients eligible for clinical trials. These results have to be confirmed in a larger independent population, in a multicentre setting, and ideally in a prospective study.

## Patients and Methods

### Population

Patients with NEC were identified from our neuroendocrine registry (ENETS centre of excellence), including gastroenteropancreatic and unknown location of primary tumours, and diagnosed between December 2002 and December 2015. The diagnosis of NEC was confirmed according to WHO 2010 classification with: i) a poorly differentiated carcinoma (small-cell or large-cell), ii) a grade 3 tumour (Ki-67 > 20% and/or mitotic index > 20 mitotic count per 10 high power field, HPF, (2 mm^2^)), and iii) immunohistochemical detection of at least two neuroendocrine markers including CgA and synaptophysin^23^. All pathological specimens and/or pathological charts were reviewed by pathologists of the institution and only patients without doubt as to the diagnosis were included. There were no major differences in the previous and recent WHO classifications for the diagnosis of NEC, while few minor changes in the classification of well-differentiated NET were made during the revision of the WHO classification. Our database is registered and this cohort was approved by CNIL (*Commission nationale de l’informatique et des libertés*) on 6 November 2015 (no.15–111). All methods were performed in accordance with the relevant guidelines and regulations. All patients signed an informed consent.

### Clinical, morphological and histological data

The following clinical features were recorded for all patients at diagnosis: age, gender, and presence or absence of symptoms within the 3 months before diagnosis of NEC. Weight loss was defined as the occurrence of a >5% decrease in weight within the 3 months before diagnosis. Morphological data collected included primary and metastatic locations, TNM stage, number of metastatic sites, uptake on somatostatin receptor scintigraphy (SRS, recorded as positive/negative) with indium-111-pentetreotide, and uptake on ^18^F-fluorodeoxyglucose positron emission tomography (FDG-PET, recorded as positive/negative). Pathological data included cell size (small *versus* large), Ki-67 index, and presence of necrosis. Immunohistochemistry of CgA and synaptophysin were recorded as positive and negative. Evaluation of Ki-67 expression was performed in at least 2000 tumour cells, according to the current recommendations for GEP-NET^24^. Patients were followed (clinically with thoraco-abdomino-pelvic computed-tomography scan) every 2–3 months for 2 years after the diagnosis, then every 6 months.

### Biological data

Biological data were collected in our laboratory at the time of diagnosis of NEC + /−2 weeks, and before the first treatment (chemotherapy): serum plasma levels of neuroendocrine markers CgA and NSE, LDH, liver functions tests (ALP, alanin aminotransferase – ALT, AST, total bilirubin), inflammatory markers (albumin, CRP, fibrinogen), haemostatic markers (PT, aPTT, haemoglobin – Hb, and platelets) and complete blood count (leukocytes, lymphocytes, neutrophils). The NLR was calculated and was considered high if NLR ≥ 3^7^,^8^ or ≥ 4 25. For all statistical analysis of biological data, we first analysed the median value as a cut-off. Based on the literature, CgA^4^,^23^, NSE^4^,^23^, and LDH^3^,^26^ levels were considered high if they were 2 ULN. Thus, all the factors were examined as both continuous and categorical variables (2 ULN). Substrates used and technical changes for biochemical analysis during the study period are presented in Table S1.

### Statistical analysis

Categorical variables were expressed as percentages and continuous variables were expressed as median with range. OS was calculated from the date of diagnosis to the date of death or last follow-up. OS were assessed using the Kaplan–Meier method and comparisons were performed using the log-rank test. For continuous variables, the chosen cut-off level chosen was their median value and we explored cut-offs described in the literature for Ki-67 (55%)^3^, CgA (2 ULN)^3^,^4^, NSE (2 ULN)^4^,^23^, LDH (2 ULN)^3^,^4^,^26^, and NLR (≥3 or ≥4)^7^,^8^,^25^. Only variables with a *P* value of < 0.05 according to univariate analysis were introduced in the Cox model. Relative risks were expressed as hazard ratios with 95% confidence intervals. A *P* value of < 0.05 was considered statistically significant. The cut-off date for the final analysis was 1 May 2016. All statistical analyses were performed using Statistical Package for Social Sciences version 17.0 (SPSS, Chicago, IL, US).

## Additional Information

**How to cite this article**: Freis, P. *et al*. Prognostic factors in neuroendocrine carcinoma: biological markers are more useful than histomorphological markers. *Sci. Rep.* **7**, 40609; doi: 10.1038/srep40609 (2017).

**Publisher's note:** Springer Nature remains neutral with regard to jurisdictional claims in published maps and institutional affiliations.

## Supplementary Material

Supplementary Information

## Figures and Tables

**Table 1:** Clinical data and univariate analysis of overall survival.

	n (%)	Median OS, months (95% CI)	*P* value
All patients	100 (100)	16 (13.9–18.1)	—
Age at diagnosis (years)^a^	64 (30–89)	—	0.26
Gender: male	67 (67)	—	0.74
Primary tumour location (n = 100)			0.88
Unknown	24 (24)	15 (11.9–18.1)	
Oesophagus	4 (4)	16 (0.0–38.5)	
Stomach	11 (11)	16 (3.6–28.4)	
Duodenum	7 (7)	14 (0.0–28.4)	
Pancreas	23 (23)	19 (12.3–25.7)	
Jejunum	1 (1)	11 (−)	
Colon	10 (10)	12 (3.2–20.8)	
Rectum	16 (16)	16 (3.5–28.5)	
Anal canal	2 (2)	18 (−)	
Other^b^	2 (2)	16 (11.7–20.3)	
Clinical symptoms at diagnosis (n = 89)			
Pain	51 (57)	—	0.96
Weight loss	36 (40)	—	0.52
Bowel disorders	18 (20)	—	0.41
Icterus	12 (13)	—	0.16
Mass syndrome	5 (6)	—	0.76
ECOG-PS (n = 64)			0.02
<2	44 (69)	18 (14.0–22.0)	
≥2	20 (31)	9 (7.0–11.0)	
Functioning tumours (n = 94)	1, ACTH (1)	—	0.23
Stage (n = 100)			0.02
I–III (without metastasis)	19 (19)	25 (9.9–40.1)	
IV (with metastasis)	81 (81)	14 (11.5–16.5)	
Number of metastatic sites (n = 100)			0.11
0	19 (19)	25 (9.9–40.1)	
1	42 (42)	14 (12.4–15.6)	
2	22 (22)	12 (0.0–25.4)	
>2	17 (17)	12 (8.5–15.5)	
Location of metastatic sites (n = 100)			
Liver	56 (56)	—	0.11
Lymph nodes	50 (50)	—	0.71
Bone	11 (11)	—	0.99
Lung	11 (11)	—	0.18
Peritoneal	6 (6)	—	0.47
Adrenal gland	4 (4)	—	0.64
Brain	4 (4)	—	0.28
Other^c^	7 (7)	—	0.88
Nuclear imaging			
SRS uptake (n = 24)	11 (48)	—	0.11
FDG-PET uptake (n = 46)	42 (91)	—	0.33
Primary tumour resection (n = 100)			0.004
Yes	28 (28)	25 (14.1–35.9)	
No	72 (72)	13 (11.4–14.6)	
Metastasis resection (n = 100)			0.32
Yes	11 (11)	27 (8.5–45.5)	
No	89 (89)	15 (12.6–17.4)	

Abbreviations: ACTH, Adrenocorticotropic hormone; CI, confidance interval; ECOG-PS, Eastern Cooperative Oncology Group Performance status; FDG-PET, fluorodeoxyglucose positron emission tomography; OS, overall survival; SRS, Somatostatin receptor scintigraphy; Notes: ^a^Median (range); ^b^Other primary tumor sites: appendix (1), gallbladder (1); ^c^Other locations of metastasis: kidney (1), muscle (1), cutaneous (2), pancreas (3), thyroid gland (1), breast (1), ovary (1).

**Table 2:** Biological data and univariate analysis of overall survival (the cut-off was the median value of each marker or twice the upper limit of normal when specified).

	Median (range)	Median OS, months (95% CI)	*P* value
CgA, μg/L (n = 44)	156.5 (20.0–11192.0)	—	0.64
CgA < 2 ULN^a^	26 (59)	16 (14.1–17.9)	0.85
CgA ≥ 2 ULN^a^	18 (41)	14 (9.4–18.6)	
NSE, μg/L (n = 44)	34.1 (6.6–956.1)		0.12
NSE < 2 ULN^a^	18 (41)	17 (13.6–20.4)	0.13
NSE ≥ 2 ULN^a^	26 (59)	13 (8.1–17.9)	
LDH, IU/L (n = 52)	338 (130–2722)	—	0.33
LDH < 2 ULN^a^	37 (71)	19 (13.4–24.6)	<0.001
LDH ≥ 2 ULN^a^	15 (29)	9 (8.0–10.0)	
ALP, IU/L (n = 59)	141 (30–2362)	—	0.64
ALT, IU/L (n = 58)	29 (6–487)	—	0.55
AST, IU/L (n = 61)	29.5 (7.0–376.0)	—	0.58
AST < 2 ULN^a^	42 (69)	18 (13.1–22.9)	0.001
AST ≥ 2 ULN^a^	19 (31)	8 (0.9–15.1)	
Total bilirubin, μmol/L (n = 58)	9.5 (2.0–493.0)	—	0.74
Albumin, g/L (n = 52)	37.9 (20.3–45.0)	—	0.58
CRP, mg/L (n = 53)	18.9 (0.6–224.5)	—	0.29
Fibrinogen, g/L (n = 53)	4.5 (1.9–7.4)	—	0.24
PT, % (n = 55)	96 (20–111)	—	0.29
aPTT, ratio (n = 47)	1.0 (0.8–3.9)	—	0.87
Hb, g/L (n = 63)	126.0 (49.1–159.0)	—	0.86
Leukocytes, G/L (n = 63)	7.4 (2.1–27.2)	—	0.66
Lymphocytes, G/L (n = 61)	1.5 (0.5–6.0)	—	0.10
Neutrophils, G/L (n = 61)	5.5 (0.4–23.9)	—	0.85
Neutrophil/Lymphocyte ratio (n = 61)	3.6 (0.2–21.7)	—	0.16
NLR < 3^a^	23 (38)	16 (8.0–24.0)	0.35
NLR ≥ 3^a^	38 (62)	16 (13.5–18.5)	
NLR < 4^a^	34 (56)	16 (7.7–24.3)	0.06
NLR ≥ 4^a^	27 (44)	16 (13.2–18.8)	
Platelets, G/L (n = 63)	273 (81–816)		0.85

Abbreviations: aPTT, activated partial thromboplastin time; AST, aspartate aminotransferase; ALP, alkaline phosphatase; ALT, alanin aminotransferase; CgA, chromogranin A; CI, confidence interval; CRP, C-reactive protein; Hb, hemoglobin; LDH, lactate dehydrogenase; NLR, neutrophil-to-lymphocyte ratio; NSE, Neuron-specific enolase; OS, overall survival; PT, prothrombin time, ULN, upper limit of normal; Notes: ^a^n (%).

**Table 3:** Histological data and univariate analysis of overall survival.

	n (%)	Median OS, months (95% CI)	*P* value
Morphological size (n = 82)			0.79
Small cell	34 (41)	16 (8.5–23.5)	
Large cell	48 (59)	15 (13.0–17.0)	
Synaptophysin staining (n = 81)			0.12
Positive	79 (98)	16 (13.5–18.5)	
Negative	2 (2)	6 (−)	
CgA staining (n = 92)			0.97
Positive	66 (72)	16 (12.8–19.2)	
Negative	26 (28)	13 (10.6–15.4)	
Ki-67 (n = 89)^a^	70 (20–100)	—	0.58
<55%	23 (26)	22 (10.2–33.8)	0.06
≥55%	66 (74)	14 (12.0–16.0)	
Presence of necrosis (n = 58)			0.08
Yes	50 (86)	14 (11.0–17.0)	
No	8 (14)	Not reached	

Abbreviations: CgA, chromogranin A; CI, confidence interval; OS, overall survival; Notes: ^a^Median (range).

**Table 4:** Multivariate Cox regression analysis of the prognosis factors in patients with GEP-NEC.

	Hazard ratio (95% CI)	*P* value
ECOG-PS, <2 vs. ≥ 2	0.82 (0.22–3.06)	0.77
Stage, I-III vs. IV	0.75 (0.25–2.27)	0.62
LDH, <2 ULN vs. ≥ 2 ULN	0.36 (0.13–0.97)	0.04
AST, <2 ULN vs. ≥ 2 ULN	0.31 (0.11–0.91)	0.03

Abbreviations: AST, aspartate aminotransferase; CI, confidence interval; ECOG-PS, Eastern Cooperative Oncology Group Performance status; LDH, lactate dehydrogenase; ULN, upper limit of normal.
