# Supplementary material for: Prognostic factors in neuroendocrine carcinoma: biological markers are more useful than histomorphological markers
Source: Sci Rep. 2017 Jan 11;7:40609. doi: 10.1038/srep40609 (PMC5225444; doi:10.1038/srep40609)
Supplement: Supplementary Information [file srep40609-s1.pdf]

Prognostic factors in neuroendocrine carcinoma: biological markers are more useful than histomorphological markers

Patricia Freis[1,5,6], Emmanuelle Graillot[2], Pascal Rousset[3], Valérie Hervieu[1], Laurence Chardon[4], Catherine Lombard-Bohas[2], Thomas Walter[2,5]

Supplementary Table 1. Assessment of biochemical analysis.

		Technical or Substrate	Unit	Upper limit of normal
**CgA**	CisBio, 2004-2016	radioimmunoassay	µg/L	98
**NSE**	Thermofisher Brahms (Kryptor), 2003-2016	immunofluorescent assay	µg/L	12.5
**LDH**	Roche (Modular), 2002-2011<br>Siemens (Vista), 2011-2016*	lactate (IFCC recommended)	IU/L	241
**ASAT**	Roche (Modular), 2002-2011<br>Siemens (Vista), 2011-2016*	with pyridoxal 5 phosphate (IFCC recommended)	IU/L	35
**ALAT**	Roche (Modular), 2002-2011<br>Siemens (Vista), 2011-2016*	with pyridoxal 5 phosphate (IFCC recommended)	IU/L	45
**ALP**	Roche (Modular), 2002-2011<br>Siemens (Vista), 2011-2016*	p-nitrophenyl phosphate	IU/L	136
**Total Bilirubin**	Roche (Modular), 2002-2011<br>Siemens (Vista), 2011-2016*	diazonium salt (phenyldiazonium)<br>diazonium salt (sulfanilic acid)	µmol/L	17
**CRP**	Roche (Modular), 2002-2011<br>Siemens (Vista), 2011-2016*	turbidimetry<br>nephelometry	mg/L	3
**Albumin**	Roche (Modular), 2002-2011<br>Siemens (Vista), 2011-2016*	turbidimetry<br>nephelometry	g/L	35#

For albumin, we indicated the lower (not the upper) limit of the normal

* There is no statistical difference between these processes, as they are based on the same method with the same substrate, and transferability was checked before each method evolution. We used the recommended method of the International Federation of Clinical Chemistry and Laboratory Medicine (IFCC).

Abbreviations: CgA, chromogranin A; NSE, Neuron-specific enolase; LDH, lactate dehydrogenase; AST, aspartate aminotransferase; ALP, alkaline phosphatase; ALT, alanin aminotransferase; CRP, C-reactive protein.
